# Estimated Prevalence of Caprine Paratuberculosis in Boer Goat Herds in Missouri, USA

**DOI:** 10.1155/2012/674085

**Published:** 2012-11-28

**Authors:** Patrick Pithua, Nathaniel S. Kollias

**Affiliations:** Department of Veterinary Medicine and Surgery, University of Missouri, Columbia, MO 65211, USA

## Abstract

The objective of this study was to estimate true animal-level and herd-level prevalence of *Mycobacterium avium* subsp. *paratuberculosis* (MAP) antibodies in Missouri Boer goat herds. Sera harvested from blood samples collected from goats ≥24 months of age in 25 Missouri Boer goat herds were tested for presence of MAP antibodies using a commercial ELISA kit. Herds were declared positive for MAP if one or more goats in the herd tested positive for MAP antibodies. True animal, within-herd, and between-herd prevalences were calculated using the Rogan-Gladen estimator and were 1.4% (95% CI = 0.1 to 3.6%), 3% (95% CI = 0 to 6%), and 54.7% (95% CI = 28.2 to 86.2%), respectively. Findings in this study confirmed that MAP infection is endemic in Missouri Boer goat herds.

## 1. Introduction 

Paratuberculosis (PTB) is a progressive, debilitating, and production limiting disease of ruminants caused by *Mycobacterium avium* subsp. *paratuberculosis* (MAP) infection. Paratuberculosis is recognized worldwide as one of the most economically important food animal diseases affecting cattle, sheep, and goats. Recognized herd losses attributable to PTB include increased mortality and premature culling risks, lower reproductive efficiency, compromised growth rates, and decreased milk yield [1–4]. Although MAP's zoonotic potential is a subject of debate, [5, 6] the organism's ability to contaminate milk [7] plus its frequent detection in patients with Crohn's disease [8, 9] raises concern for a potential public health hazard. 

Even though goats are considered a minor species in the US, the goat industry is recognized as one of the fastest growing US livestock sectors [10]. However, caprine PTB has not received much attention in the US compared with the degree of attention that bovine PTB has received in recent years. No studies have been conducted to provide valid estimates of prevalence of MAP infection in US goat herds, although a 2009 United States Department of Agriculture Plant and Animal Health Inspection Service (USDA-APHIS) survey revealed that 1.7% of goat operations had reported suspected clinical cases of caprine PTB in 45% of these suspect herds [11]. Thus caprine PTB may be endemic, possibly widespread, and could constitute a serious problem for US goat producers. 

Valid estimates of prevalence of MAP infection in goats at both the animal and herd level are needed by industry stakeholders to determine whether the disease warrants interventions to mitigate its negative impact on herd profitability. In MAP affected goat herds, possible intervention goals could include eradication efforts in the event of very low prevalence, institution of a long-term risk-based control program that emphasizes management changes in high prevalence herds, and surveillance in the event of likely absence of infection. The objective of this study was to estimate true animal, within-herd, and between-herd prevalences of MAP antibodies in Missouri Boer goat herds. 

## 2. Materials and Methods

### 2.1. Study Design

Herd prevalence of MAP infection in Boer goat herds in the state of Missouri was determined using a cross-sectional study approved by the Institutional Animal Use and Care Committee at the University of Missouri (Protocol no. 7395). 

### 2.2. Calculations of Number of Herds and Animals to Sample

The number of herds required to determine the apparent herd prevalence of MAP infected Boer goat herds was calculated as follows [12]:

(1) $$\begin{array}{c} N_{\text{herds}}=\frac{Z_{\alpha }^{2}P_{\text{herd}}\left(1-P_{\text{herd}}\right)}{e^{2}}, \end{array}$$

where, *N*_herds_ = minimum number of herds to sample, *α* = type I error rate (assumed to be .05), *P*_herd_ = assumed prevalence of MAP positive herds (i.e., ≥0.2), and *e* = maximum allowable error. On this basis, 61 herds were required to obtain apparent herd prevalence for MAP ≥ 20% assuming an allowable error of 10% and a 5% type I error rate, although ultimately only 25 (41%) herds were studied due to low farmer response rates. Consequently, all eligible animals (i.e., Boer goats ≥ 24 months old) present in herds that agreed to the study were tested for MAP. 

### 2.3. Herds and Animal Selection Criteria

Boer goat herds in the state of Missouri constituted the sampling unit. Herds that contained only Boer goats were eligible to participate in the study. Consequently, herds with multiple breeds of goats were excluded from the study. 

The target population included Boer goats 24 months of age and older. Due to the long incubation period of PTB in ruminants and the well-recognized low sensitivity of ELISA (including the ELISA used for this study) tests for detecting MAP antibodies in nonfecal shedding and younger animals [13], goats < 24 months of age were excluded from this sero-survey. 

Prior to the study onset, herd addresses, and owner contacts were obtained from the membership list of the Missouri Meat Goat Producers Association. Based on the above inclusion criteria, a total 142 Boer goat herds in Missouri were determined to be eligible and were contacted with the request to participate in the study. Twenty-five (~18%) herd owners agreed to testing their herds for MAP representing 41% (25 of 61) of the estimated number of herds required to determine between-herd prevalence (see sample size calculations above). This relatively low response rate was expected given the sensitivity attached to data regarding MAP herd status by many producers in Missouri plus the voluntary nature of participation. 

**Figure 1:**
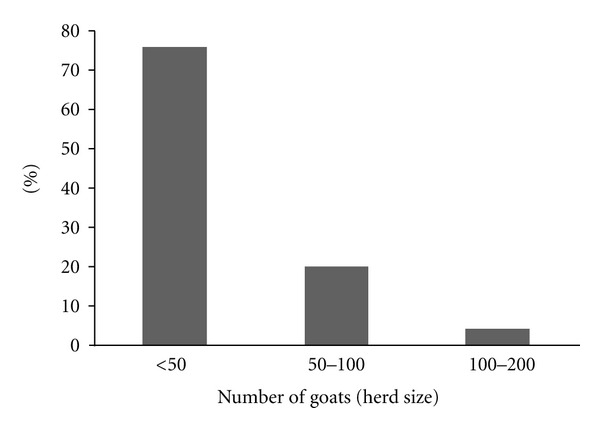


Consequently, all eligible animals (i.e., Boer goats ≥ 24 months old) present in agreeing herds were tested for MAP. Herd visits were completed between May and September, 2012. In total, 629 goats from 25 herds were tested for MAP antibodies. The mean ± SD (minimum, maximum) number of animals tested per herd was 25 ± 19 (2, 57). The proportion of herds tested that had <50, 50–100 and 100–200 goats were 76%, 20%, and 4%, respectively (Figure 1). 

### 2.4. Blood Collection

Blood samples were collected for serology from all goats that met the inclusion criteria on a single scheduled visit to each participating herd via jugular venipuncture using plain 10 mL Vacutainer (Becton, Dickinson and Co., Franklin Lakes, NJ, USA) tubes. Samples were transported to the University of Missouri's Veterinary Diagnostic Laboratory for further processing. 

### 2.5. ELISA Analysis

Blood samples were initially centrifuged for 5 minutes at 3,000 g. Sera were tested for MAP antibodies using a solid phase indirect enzyme immunoassay (Parachek, Johne's Absorbed EIA, Prionics USA, Inc.) according to the manufacturer's instructions. Presence of MAP antibodies in a sample was indicated by a sample absorbance value of 0.27 as the positive cut point [14], otherwise a negative result was declared. 

### 2.6. Data Analysis

#### 2.6.1. Case Definitions

A goat that tested positive for MAP antibodies using the Parachek, Johne's Absorbed ELISA (Prionics USA, Inc.) was considered infected. Herds were declared positive for MAP if one or more goats from the herd tested positive for MAP antibodies on the Parachek, Johne's absorbed ELISA (Prionics USA, Inc.). 

#### 2.6.2. Calculation of Apparent Prevalence

The apparent animal, within-herd, and between-herd prevalences were calculated by dividing the number of test positive outcomes by the corresponding denominator (i.e., total number of goats tested from all herds, total number of goats tested within each herd, and total number of herds tested, resp.) for each measure as described [12]. The 95% confidence intervals for apparent prevalences were estimated using the Wilson binomial approximation method as described [15]. 

#### 2.6.3. Calculation of True Prevalence

True animal, within-herd, and between-herd prevalences were calculated using the Rogan-Gladen estimator [16]. Blaker's exact confidence limits for the true prevalence estimates were calculated using a previously described method [15]. 

In all calculations, the apparent sensitivity and specificity of the Parachek, Johne's absorbed ELISA (Prionics USA, Inc.) for detecting MAP antibodies in goat sera were assumed to be 65% (range 65–88%) and 99%, respectively, based on a previous comparative study [17] against an Agar-gel immunodiffusion (AGID) assay and reported by the kit manufacturer [18]. 

## 3. Results and Discussion

In total, 12 of the 629 goats originating from the 25 herds tested were positive for MAP antibodies. Nine of the 25 herds tested had at least one MAP positive goat and were declared infected with MAP. 

The animal, within-herd, and between-herd apparent prevalences were 1.9% (95% CI = 1.1 to 3.3%), 2% (95% CI = 0 to 4%), and 36% (95% CI = 20.2 to 55.5%), respectively (Table 1). Related estimates reported in a German study of dairy goat flocks were 21%, 32%, and 71%, respectively [19]. The apparent prevalences estimated in our study are not directly comparable to the German findings given differences between the two studies in the diagnostics tests used, sampling designs, study and target populations as well as analytical methods employed [20, 21]. 

Interestingly, the true between-herd (54.7%; 95% CI = 28.2 to 86.2%) prevalence for MAP in this study was similar to related estimates reported for Cyprian (55.2%; 95% CI = 45.3 to 64.7%) and French (50%; 95% CI = 39 to 62%) dairy goat herds, respectively [21, 22]. However, the true animal (1.4%; 95% CI = 0.1 to 3.6%) and within-herd (3%; 95% CI = 0 to 6%) prevalences reported here (Table 1) were lower than those reported in studies conducted elsewhere. For example, recent studies in Cyprian [22] and French [21] dairy goat herds found a 7.9% (95% CI = 7.2 to 8.7%) and 11.1% (95% CI = 1.1 to 33.1%) true within-herd MAP prevalence, respectively. In those same populations, the true animal prevalences were 5.5% (95% CI = 5.1 to 5.9%) and 11%. 

A possible reason for the above differences in true animal and within-herd prevalences estimates could be due to a breed predisposition to MAP infection with apparent risks being greater for dairy breeds of goats than meat breeds (i.e., Boer goats), although other herd factors cannot be discounted. In cattle, herds composed of predominantly Jersey breed were more likely to be infected with MAP than those herds in which other breeds predominated [23, 24]. Studies in England found a significantly greater prevalence of MAP infection in dairy breeds of cattle compared to beef breeds [25]. While no plausible explanation exists in support of the breed-susceptibility hypothesis, increased level of exposure, due to perhaps a high within herd MAP prevalence rather than increased genetic or breed predisposition, may have been responsible for the apparently higher prevalence of infection in the dairy breeds in these studies. In an earlier study, the majority of Jersey cows that tested positive for MAP were shown to have originated from herds with greater prevalence of PTB suggesting that effect of the herd may have confounded the apparent effect of breed [23]. 

Other than the possibility of apparent breed susceptibility and the confounding effects by unrelated herd factors, a plausible reason in herd prevalence could be differences in the mobility patterns of dairy versus meat goats. For example, in dairy goat management, some goats may be moved to other farms over their lifetime as owners buy in or sell out animals. This apparent between-herds mobility is more likely a dairy goat phenomenon and may explain the higher animal and herd prevalences of MAP infection in dairy relative to the meat breeds of goats. 

In this study, the Rogan-Gladen [16] estimator was used to estimate true MAP prevalence while adjusting for the assumed 65% apparent sensitivity and 99% specificity of the Parachek, Johne's Absorbed ELISA (Prionics USA, Inc.) kit for detecting MAP antibodies in goat sera. While this approach provided a single true MAP prevalence estimate for each herd, the estimates obtained tended to be meaningless (i.e., true herd prevalence estimates <0%) for herds with zero percent apparent within-herd MAP prevalence (data not shown). This was to be expected given that, in the majority of the herds studied, sample-sizes were small (mean ± SD; minimum, maximum; 25 ± 19; 2, 57) and MAP prevalences may have been low. In addition, the preceding sensitivity and specificity estimates were based on results obtained in a previous study comparing the current absorbed ELISA against an AGID test for detecting MAP antibodies in goats known to have PTB [17]. The latter study was therefore not a classical diagnostic test validation effort. Thus, the reported sensitivity (i.e., 65%) was likely a gross overestimate of the true sensitivity of the Parachek, Johne's absorbed ELISA (Prionics USA, Inc.) used in this study, given that the study population comprised 19 goats known to have PTB. Regardless, we used this test because the manufacturer recommends it's use in epidemiological studies and the management and control of PTB in cattle, sheep, and goats [18]. While the adoption of a Bayesian approach [26] could have resolved this issue, we, nonetheless, chose to use the Rogan-Gladen estimator to calculate the true prevalence estimates since the application of the Bayesian approach was beyond the scope of this publication [17, 20]. 

Finally, the results presented here must be interpreted cautiously given some obvious study limitations. First, given that the apparent sensitivity (i.e., 65%) of the assay used in the current study possibly represented an overestimate of the true sensitivity of the Parachek, Johne's absorbed ELISA (Prionics USA, Inc.) for detecting MAP antibodies in caprine sera, the true prevalence estimates reported here may be an underestimate of the true MAP prevalence in Missouri Boer goat herds. Second, of the 61 herds initially required to estimate herd prevalence, only 25 (41%) agreed to be tested for MAP in this study. This limited number of participating herds coupled with the larger percentage of smaller herds (Figure 1) agreeing to testing in addition to the low numbers of animals tested per herd (mean ± SD; minimum, maximum; 25 ± 19; 2, 57), meant that the calculated prevalence estimates were statistically unstable. It is therefore, not surprising that the confidence intervals associated with the MAP prevalence estimates are wide (Table 1). Third, the extent to which these study's results can be extrapolated to a wider population is undermined by the focus on a single breed of goats originating from only one state. Therefore, while these prevalence estimates may reflect the status of MAP in Boer goat herds in Missouri, these findings should not be construed as indicative of the national burden of caprine PTB in other breeds of goats reared in the US. 

## 4. Conclusions

To our knowledge, this is the first study performed in Missouri to quantify prevalence of MAP infection in a caprine population. Findings in this study confirmed that MAP infection is endemic in Missouri Boer goat herds. With an estimated meat goat population of approximately 37,151 head [27] in the state, it is likely that 520 (95% CI = 37 to 1337) of Missouri's goat population is infected with MAP. 

Future studies are warranted to further validate current screening tests for MAP antibodies in caprine sera and to characterize specific risk factors associated with MAP prevalence in Missouri (and indeed US) goat herds in order to understand the specific impact of caprine PTB on profitability and performance levels of both meat and dairy goat enterprises under the current US goat production systems.

## Figures and Tables

**Table 1:** Apparent and true prevalence estimates for animal, within-herd, and between-herd prevalences derived from 629 Boer goats in 25 Missouri herds.

Prevalence type	Number tested	Number positive for MAP	Apparent prevalence		True prevalence	
			Estimate, %	95% CI	Estimate, %	95% CI
Animal	629	12	1.9	1.1 to 3.3	1.4	0.1 to 3.6
Within-herd	—	—	2	0 to 4	3	0 to 6
Between-herd	25	9	36	20.2 to 55.5	54.7	28.2 to 86.2
